# Thrombospondin 1 is a key mediator of transforming growth factor β-mediated cell contractility in systemic sclerosis via a mitogen-activated protein kinase kinase (MEK)/extracellular signal-regulated kinase (ERK)-dependent mechanism

**DOI:** 10.1186/1755-1536-4-9

**Published:** 2011-03-31

**Authors:** Yunliang Chen, Andrew Leask, David J Abraham, Laura Kennedy, Xu Shi-wen, Christopher P Denton, Carol M Black, Liaquat S Verjee, Mark Eastwood

**Affiliations:** 1 School of Life Sciences, University of Westminster, London, UK; 2 Canadian Institute of Health Research Group in Skeletal Development and Remodelling, Division of Oral Biology and Department of Physiology and Pharmacology, Schulich School of Dentistry, University of Western Ontario, London, Ontario, Canada; 3 Department of Inflammation, Centre for Rheumatology, University College London, London, UK; 4 Kennedy Institute of Rheumatology, Imperial College London, London, UK

## Abstract

**Background:**

The mechanism underlying the ability of fibroblasts to contract a collagen gel matrix is largely unknown. Fibroblasts from scarred (lesional) areas of patients with the fibrotic disease scleroderma show enhanced ability to contract collagen relative to healthy fibroblasts. Thrombospondin 1 (TSP1), an activator of latent transforming growth factor (TGF)β, is overexpressed by scleroderma fibroblasts. In this report we investigate whether activation of latent TGFβ by TSP1 plays a key role in matrix contraction by normal and scleroderma fibroblasts.

**Methods:**

We use the fibroblast populated collagen lattices (FPCL) model of matrix contraction to show that interfering with TSP1/TGFβ binding and knockdown of TSP1 expression suppressed the contractile ability of normal and scleroderma fibroblasts basally and in response to TGFβ. Previously, we have shown that ras/mitogen-activated protein kinase kinase (MEK)/extracellular signal-regulated kinase (ERK) mediates matrix contraction basally and in response to TGFβ.

**Results:**

During mechanical stimulation in the FPCL system, using a multistation tensioning-culture force monitor (mst-CFM), TSP1 expression and p-ERK activation in fibroblasts are enhanced. Inhibiting TSP1 activity reduced the elevated activation of MEK/ERK and expression of key fibrogenic proteins. TSP1 also blocked platelet-derived growth factor (PDGF)-induced contractile activity and MEK/ERK activation.

**Conclusions:**

TSP1 is a key mediator of matrix contraction of normal and systemic sclerosis fibroblasts, via MEK/ERK.

## Background

Scleroderma (systemic sclerosis (SSc)) is a chronic disease of unknown aetiology characterised by microvascular injury, autoimmune inflammatory responses, and severe and often progressive fibrosis [1-3]. There is no therapy for the fibrosis observed in SSc. SSc dermal fibroblasts can be isolated and cultured readily, and will retain their enhanced expression of type I collagen and α smooth muscle actin, (α-SMA) [4-7]. Thus, examination of the molecular difference that may exist between normal fibroblasts from healthy individuals and fibroblasts from 'lesional' areas of SSc patients would seem to be an ideal system to yield valuable insights into the pathogenesis of SSc. Although the molecular basis for SSc is unclear, we have previously shown that fibroblast from scarred (lesional) area of SSc patients show elevated constitutive extracellular signal-regulated kinase (ERK) activation and overexpress a cohort of profibrotic genes including connective tissue growth factor (CTGF, also known as CCN2), and the heparan sulfate containing proteoglycans (HSPGs) syndecan 2 and syndecan 4 [7,8]. As one of the extracellular modular glycoproteins, thrombospondin (TSP)1 was also found to be highly expressed in SSc dermal fibroblasts [9]. Significantly, whereas non-lesional and lesional SSc fibroblasts produce similar amounts of type I collagen, lesional SSc fibroblasts show markedly enhanced abilities to adhere to and contract extracellular matrix [7]. The enhanced contractile ability of lesional SSc fibroblasts was suppressed by blocking HSPG biosynthesis, mitogen-activated protein kinase kinase (MEK) or antagonising transforming growth factor (TGF)β receptor type I (activin-linked kinase 5 (ALK5)) [7,10]. Enhanced activation of ERK was also observed in lesional SSc [7]. Moreover, heparan sulfate-dependent ERK activation contributes to the overexpression of profibrotic proteins and the enhanced contraction by lesional dermal scleroderma fibroblasts of their extracellular matrix [11]. We have begun to dissect the role that individual proteins play in fibroblast activation; for example, the HSPG syndecan 4 is required both for basal and growth factor-induced ERK activation in normal fibroblasts and for the enhanced activation of ERK observed in lesional SSc fibroblasts [7]. However, overall, the fundamental roles of individual matrix proteins in SSc pathogenesis are largely unknown.

TGFβ has long been hypothesised to be a major contributor to pathological fibrotic diseases. As TGFβ induces fibroblasts to synthesise and contract the extracellular matrix (ECM), this cytokine has long been believed to be a central mediator in wound healing and fibrotic responses, including SSc [12]. Despite the fact that enhanced ECM contraction and adhesion observed in SSc fibroblasts depends on TGFβ type I receptor (ALK5) activity, the fundamental mechanism underlying the contribution of TGFβ to the fibrotic phenotype of SSc is unclear as, in this cell type, ALK5 inhibition was unable to reduce critical features of the myofibroblast phenotype, such as α-SMA expression and stress fibre formation [10]. The majority of the studies conducted thus far has measured acute responses to TGFβ and suggest that TGFβ alone is insufficient for sustained fibrogenic responses [12,13]. Recently, we have shown that TGFβ signalling partially contributes to the fibrotic phenotype of SSc fibroblasts, resulting from an exaggeration of processes normally operating in cells [7,10]. However, so far relatively little is known about the underlying cause of this exaggerated TGFβ signalling and how this might contribute to the enhanced contractile activity of SSc lesional fibroblasts.

TSP1, an extracellular modular glycoprotein secreted by many cell types, is a component of the extracellular matrix in remodelling tissues and can bind to different matrix proteins and cell surface receptors, including proteoglycans, non-integrin, and integrin receptors [14]. The latter include α3β1 and α5β3 integrin receptors [15]. TSP1 also interacts with structural proteins such as collagens, fibronectin, and laminins. These interactions may present TSP1 to the cell surface, where it can mediate interactions between these proteins and their receptors [14]. These abilities account for multifunctional nature and sometimes contradictory functions of TSP1, which include influencing platelet function, angiogenesis, tumour biology, wound healing, and vascular disease [16]. TSP1 may execute many of its functions through its ability to activate TGFβ *in vitro *and *in vivo*. TSP1 binds the latency-associated peptide (LAP) of the latent TGFβ complex. Thrombospondin-LAP complex formation involves the activation sequence of thrombospondin 1 (KRFK) and a sequence (LSKL) near the N-terminus of LAP that is conserved in TGFβ [1-5]. The interactions of LAP with TSP1 through the LSKL and KRFK sequences are important for thrombospondin-mediated activation of latent TGFβ, since LSKL peptides competitively inhibit latent TGFβ activation by TSP1 or other KRFK-containing peptides [17]. Providing evidence of functional relevance of these observations to fibrotic diseases, such as SSc, recombinant TSP1 promotes fibroblast-mediated floating collagen gel contraction induced by TGFβ [18].

Consequently, much interest exists, from both clinical and pharmaceutical points of view, in identifying not only whether TSP1 can promote the pathogenesis of fibrotic diseases such SSc, but also whether targeting TGFβ signalling by antagonising TSP1 might be useful for treating these disorders. In this study, we hypothesised that TSP1 may be an endogenous activator of TGFβ during contraction of extracellular matrix in normal and SSc fibroblasts. We used the fibroblast populated collagen lattices (FPCL) system of matrix contraction to evaluate the contribution of TSP1 to the contractile activity of normal and SSc fibroblasts both basally and in response to TGFβ. We show that using TSP1 blocking peptide, or small interfering (siRNA) recognising TSP1, affects the contractile activity of normal and SSc fibroblasts. Our results provide novel insights into the underlying mechanisms behind matrix contraction by fibroblasts and the exaggerated TGFβ signalling observed in the pathogenesis of SSc.

## Methods

### Cell culture

Briefly, cell culture was performed as previously described [19,20]. Dermal fibroblasts from lesional (clinically affected) areas of female patients with diffuse SSc (duration of between 12 and 18 months) and normal individuals were taken from biopsies of age, sex and anatomically site-matched volunteers, after informed consent and ethical approval was obtained. All patients fulfilled the criteria of the American College of Rheumatology for the diagnosis of diffuse SSc, as defined by LeRoy *et al. *[21]. Fibroblasts were maintained in Dulbecco's modified Eagle medium (DMEM) (First Link, Birmingham, UK), 10% foetal bovine serum (First Link, Birmingham, UK), 100 U/ml penicillin, and 100 mg/ml streptomycin, 5% CO_2_. Fibroblasts were subcultured 1:4 at confluence. When appropriate, TSP1 blocking peptides (LSKL and an inert control peptide SLLK), ERK inhibitor U0126 (10 μM, Promega), the ALK5 inhibitor SB 431542 (10 μM, Tocris, Bristol, UK), platelet-derived growth factor (PDGF) receptor inhibitor Gleevec (2 mM, imatinib mesylate), or interferon (IFN)β (5 ng/ml, Serono Pharmaceutical Research Institute, Geneva, Switzerland) was added.

### Western blot and immunofluorescence analysis

Fibroblasts within three-dimensional collagen matrices following FPCL contraction or fibroblasts from monolayer culture were collected and lysed with 8 M urea and 1% SDS sample buffer. Proteins were quantified (Bradford, Bio-Rad, Hercules, California, USA), and equal amounts of protein (25 μg) were subjected to SDS/PAGE using 4% to 12% polyacrylamide gels (Invitrogen, Paisley, UK). Gels were blotted onto nitrocellulose, and proteins were detected using anti-CCN2 (Santa Cruz, Wembly, UK), anti α-SMA (Sigma, St Louis USA), anti-syndecan 4, anti-α3 and anti-β5 integrin (Zymed, Paisley, UK), anti-thrombospondin 1 (TSP1) (Abcam, Cambridge, UK), anti α-SMA (Sigma, St Louis USA), anti p-SMAD3 (Cell Signalling, Paisley UK) and appropriate horseradish peroxidase (HRP)-conjugated secondary antibodies (Cell Signalling, Paisley UK) and an enhanced chemiluminescence (ECL) kit (Amersham, Little Chalfont, UK). Densitometry was performed using Quantity One software (Bio-Rad, Hercules, California, USA ). For immunofluorescence detection, cells were fixed in 3% paraformaldehyde (15 min) and localisation of proteins was detected as previously described [7].

### Real-time PCR

Cells were serum-starved for 24 h and treated with or without inhibitors, as indicated, for an additional 24 h. Total RNA was isolated using Trizol (Invitrogen Paisley, UK,)and the integrity of the RNA was verified by Agilent bioanalyser. Total RNA (25 ng) was reverse transcribed and amplified using TaqMan One-step master mix and Assays on Demand primers (Applied Biosystems, Carlsbed, California, USA) in 15 μl reaction volumes with 6-carboxyfluorescein labelled TaqMan MG probe. Signals were detected using the ABI Prism 7900 HT sequence detector (Perkin-Elmer-Cetus, Vaudreuil, Canada). Triplicate samples were run, transcripts, and expression values were standardised to values obtained with control 28S RNA primers as previously described using the ΔCt method [22].

### FPCL

Measurement of contractile force generated within a three-dimensional, tethered floating fibroblast-populated collagen lattice was performed as described previously [23,24]. Using 1 × 10^6 ^cells/ml of collagen gel (First Link, Birmingham, USA), we measured the force generated across the collagen lattice with a culture force monitor (CFM). This instrument measures the minute forces exerted by cells within a collagen lattice over 24 h as fibroblasts attach, spread, migrate and differentiate into myofibroblasts [25]. In brief, a rectangular fibroblast seeded collagen gel was cast and floated in medium with 10% fetal calf serum (FCS), or in 2% FCS when the effect of antagonising TGFβ was examined. The collagen gels were tethered to two flotation bars on either side of the long edges, and, in turn attached to a ground point at one end and a force transducer at the other. Cell-generated tensional forces in the collagen gel were detected by the force transducer and logged into a personal computer. Graphical readings were produced every 15 s providing a continuous measurement of force (Dynes: 1 × 10^-5 ^N) generated [25].

Mechanical stimulation of both normal and SSc fibroblasts was achieved with the use of the multistation tensioning-culture force monitor (mst-CFM) using this system FPCLs were prepared as previously described, placed into the mst-CFM and allowed to contract endogenously for 12 h prior to a further 12 h of mechanical stimulation as previously described [26]. The cells used in these experiments were passage matched; all control and inhibition experiments were run in parallel.

### Floating collagen gel contraction assay

Experiments were performed essentially as described previously [7]. Briefly, 24-well tissue culture plates were precoated with bovine serum albumin (BSA). Normal and SSc lesional fibroblasts were treated with TGFβ or PDGF with or without ERK inhibitor U0126 (10 μM, Promega, Madison, Winsconsin, USA), the ALK5 inhibitor SB 431542 (10 μM, Tocris, Bristol, UK), the PDGF receptor inhibitor Gleevec (2 mM, imatinib mesylate), or IFNβ (5 ng/ml, Serono Pharmaceutical Research Institute SA, Geneva, Switzerland) for 24 h. Pretreated fibroblasts were suspended in MCDB medium (Sigma, Paisley UK) and mixed with collagen solution (one part 0.2 M N-2-hydroxyethylpiperazine-N'-2-ethanesulfonic acid (HEPES), pH 8.0, four parts collagen (Vitrogen-100, 3 mg/ml) and five parts of MCDB × 2), yielding a final concentration of 80,000 cells per ml and 1.2 mg/ml collagen. Collagen/cell suspension (1 ml) was added to each well. After polymerisation, gels were detached from wells by adding 1 ml of MCDB medium with PDGF, TGFβ or tumour necrosis factor (TNF)β. Contraction of the gel was quantified by loss of gel weight and decrease in gel diameter over a 24-h time period.

### siRNA knockdown

Specific siRNA recognising TSP1 was purchased as a pool of three predesigned siRNAs (siRNA#138861, siRNA#12934 and siRNA#12845) alone with a recommended control siRNA (Ambion, Warrington, UK). Normal and SSc fibroblasts were transfected using Silencer siRNA Transfection II Kit (Ambion, Applied Biosystems, Warrington UK). Cells were transfected either with control siRNA or control siRNA with TSP1 siRNA. Western blot analysis with an anti-TSP1 antibody was performed to check the efficiency of the siRNA to reduce TSP1 protein expression. The contractile ability of the cells was analysed as described above.

## Results

### Blocking TSP1 activation of latent TGFβ with LSKL peptide decreased the enhanced contractile activity of fibrotic SSc fibroblasts

**Figure 1:**
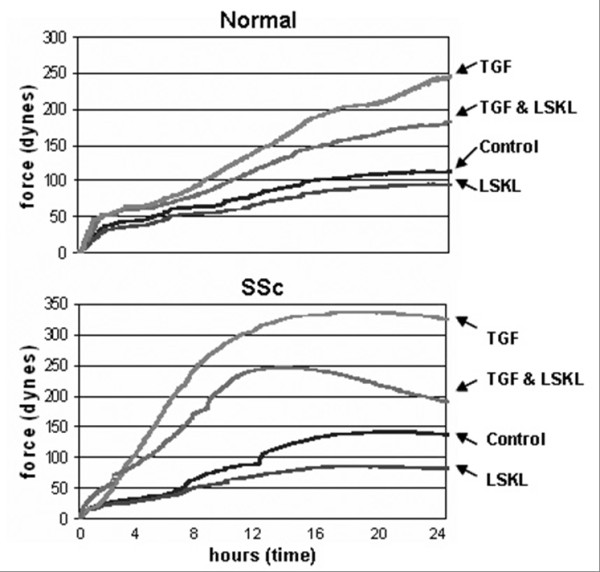
**The enhanced contractile activity of fibrotic systemic sclerosis (SSc) fibroblasts can be moderated by blocking thrombospondin 1 (TSP1) activation of latent transforming growth factor (TGF)β with a LSKL peptide**. Normal and SSc fibroblasts were pretreated for 5 days with LSKL peptide, a selective antagonist of TSP1. Cells were then suspended in a collage lattice prior to contraction in the culture force monitor (CFM). Blocking the TSP1 function with the LSKL peptide reduced the force generated by both normal and SSc cells even after treatment with TGFβ. Note that LSKL peptide also reduced the basal contractile force in normal fibroblasts groups. SLLK peptide was used as an inert control.

Both overexpression of TSP1 and elevated TGFβ activity can be found in SSc dermal fibroblasts [7,9,10]. We wanted to evaluate whether TSP1 mediates matrix contraction in fibroblasts by assessing if interfering with binding of TSP1 to TGFβ suppresses the basal and TGFβ-induced contractile activity of normal or SSc fibroblasts. LSKL peptides (selective antagonists of TSP1, which specifically interfere with the ability of TSP1 to activate TGFβ) and SLLK peptide (an inert control) [27] were used in the FPCL assay of matrix contraction. Fibroblasts in the three-dimensional FPCL system generate contractile forces, similar those found in scars and in granulation tissue undergoing matrix remodelling during normal and pathological situations [23,24,28]. Healthy and SSc fibroblasts were pretreated with TSP1 blocking peptides (LSKL) or control peptide (SLLK) for 5 days and then transferred to a culture force monitor (CFM) and forces exerted by cells within the collagen lattice over 24 h in 2% serum, both in the presence and absence of added TGFβ [27] were measured and recorded. At the end of the culture period, the LSKL peptide reduced the contractile force generated by SSc fibroblasts by about 25%, and also significantly blocked the TGFβ-induced contractile force in both normal and SSc fibroblast groups, by 24% and 41%, respectively. The LSKL peptide also showed reduced the basal contractile force generated by normal fibroblasts by approximately 14% (Figure 1). These results suggested the intriguing notion that activation of endogenous latent TGFβ played a key role in ECM contraction by both healthy and fibrotic fibroblasts.

### Blocking TSP1 activation of TGFβ with LSKL peptide impacted on the mitogen-activated protein kinase (MAPK) signalling pathways and reduced matrix protein expressions in SSc fibroblasts

**Figure 2:**
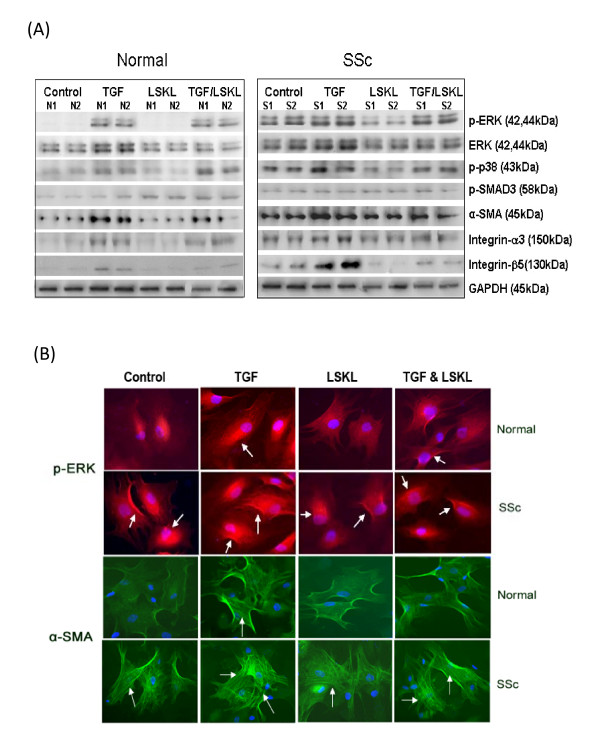
**Blocking thrombospondin 1 (TSP1) signalling with a LSKL peptide reduces matrix protein expression in both systemic sclerosis (SSc) and normal fibroblasts**. **(a) **Following contraction in the culture force monitor (CFM) the LSKL peptide-treated fibroblasts were prepared for western blot analyse to determine the protein expression levels. LSKL peptide reduced the expression of some profibrotic proteins and mitogen-activated protein kinase (MAPK) activities in SSc fibroblasts. **(b) **Immunofluorescence staining demonstrated that the LSKL peptide-treated SSc fibroblasts demonstrated a reduction in α smooth muscle actin (α-SMA) stress fibres and reduced the presence of p-extracellular signal-regulated kinase (ERK).

Lesional dermal SSc fibroblasts are characterised by the markedly enhanced ability to adhere to and contract extracellular matrix [7]. To further investigate the mechanism underlying TSP1-dependent contractile activity, fibroblasts in fully contracted FPCL gel samples (that is, 24 h post culturing in the FPCL system) were analysed by western blotting to evaluate whether in this context TSP1 blocking peptide reduced expression of matrix proteins and the activation of procontractile signalling pathways. Western blot analysis revealed that the TSP1 blocking peptide reduced expression of profibrotic proteins such as α-SMA, integrin α3, integrin β5, and the activation of p-ERK and p-p38 kinase in SSc fibroblasts (Figure 2a). Moreover, TGFβ-induced matrix gene expression and ERK and p38 phosphorylation in both normal and SSc fibroblasts were also reduced (Figure 2a).

TGFβ causes fibroblasts to differentiate into myofibroblasts, the α-SMA containing cells that are involve in the contraction processes in wound contraction and fibrosis tissue *in vivo *[24,29,30]. ERK activation contributes to the enhanced contraction by lesional dermal scleroderma fibroblasts by promoting the assembly of α-SMA stress fibres [11]. To extend our data obtained by western blot analyses indicating that LSKL peptide reduced ERK activation and α-SMA expression in SSc fibroblasts, we employed indirect immunofluorescence analysis to show that a 24-h treatment of SSc fibroblasts with LSKL peptide reduced the appearance of α-SMA stress fibres and the intense p-ERK staining, both key features characterising SSc fibroblasts, Moreover, the LSKL peptide also blocked TGFβ-induced α-SMA expression and p-ERK activity in normal and SSc fibroblasts (Figure 2b).

### TSP1 is a key mediator promoting SSc fibroblast contraction

**Figure 3:**
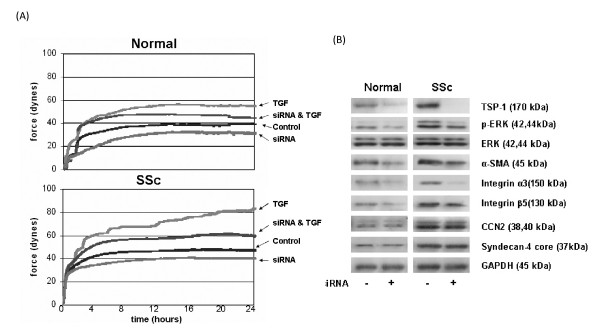
**Knockdown thrombospondin 1 (TSP1) expression through small interfering (si)RNA transfection alters the contractile characteristics and protein expression in both normal and systemic sclerosis (SSc) fibroblasts**. **(a) **The contractile ability of SSc and normal fibroblasts were reduced following siRNA transfection. **(b) **Western blot demonstrating a reduction in protein expression following siRNA transfection.

Based on the above findings, it needed to be elucidated whether TSP1 could directly mediate the enhanced contractile activities of SSc fibroblasts. To perform this analysis, we reduced TSP1 protein expression in normal and SSc fibroblasts using siRNA recognising TSP1. Western blot analysis was used to assess the ability of siRNA recognising TSP1, compared to control siRNA, to reduce TSP1 protein expression levels (Figure 3b). The contractile ability of TSP1 knockdown cells was analysed using the CFM system. We found that the contractile ability of SSc fibroblast was reduced by 16% at the 24th hourly time point after TSP1 expression knockdown; in addition, TGFβ-induced contractility of both normal and SSc fibroblasts were diminished by 18% and 29%, respectively, at the 24-h time point. The basal contractility of normal fibroblasts was reduced 19% at this time point (Figure 3a). Western blot assays were also performed with fibroblasts treated with TSP1 siRNA (Figure 3b). After TSP1 knockdown in fibroblasts from normal and SSc patients, p-ERK activation was reduced, concomitant with decreased expression of integrin α3. Consistent with prior data using an ALK5 inhibitor [10], extremely modest reduction of α-SMA and integrin β5 were observed. Expression of CCN2 and syndecan 4 was not altered in normal and SSc fibroblasts confirming previous evidence that basal expression of these proteins is independent of the TGFβ pathway [11].

### TSP1 expression and p-ERK activation were enhanced by the external mechanical force loading stimulation

**Figure 4:**
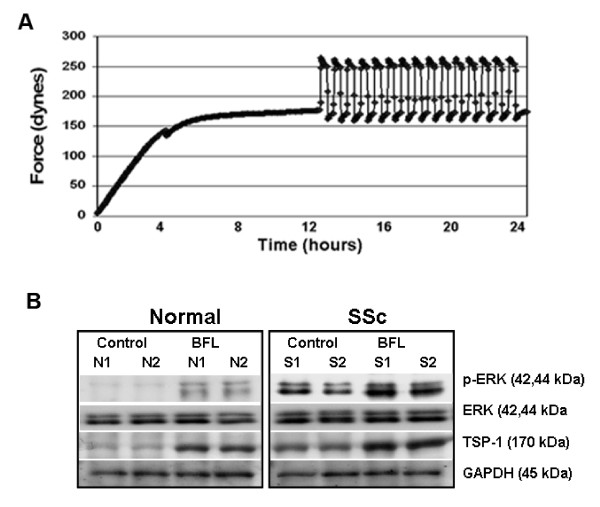
**Thrombospondin 1 (TSP1) expression and p-extracellular signal-regulated kinase (ERK) activation in fibroblasts is enhanced by applied mechanical stimulation**. **(a)**. Cells were allowed to contract the collagen matrix for 12 h prior to the application of an external load for a further 12 h. **(b) **Following gel contraction and mechanical loading cells were recovered from the gels and the levels of protein expression determined by western blot. TSP1 expression and p-ERK activation were significantly increased following mechanical stimulation in both normal and systemic sclerosis (SSc) fibroblasts.

It has been suggested that TSP1 plays a significant role in wound healing [30]. Fibroblasts loaded by biomechanical forces within the three-dimensional FPCL system remodel their matrix resulting in potent differentiation into myofibroblasts similar to that observed in wound tissue and pathological scarring [28]. As our previous data suggested that TSP1-mediated activation of TGFβ played a key role in matrix contraction by normal and fibrotic fibroblasts, we wondered if fibroblast-induced ECM contraction itself was sufficient to induce TSP1 expression. Thus, fibroblasts from normal and SSc patients were mechanically loaded to a magnitude similar to that seen in skin wounds [31-35]. During mechanical loading, cells within the FPCL system went through normal gel contraction for 12 h, after which cyclical mechanical forces were exerted on cells controlled by a computer. Each cycle consisted of force loading for 9 min followed by a 15-min resting phase prior to unloading for an additional 9 min followed by a further 15-min resting phase. Cycles were repeated 15 times for an additional 12 h (Figure 4a). ERK activation contributes to the overexpression of fibrotic proteins and the enhanced contraction by lesional dermal scleroderma fibroblasts [11]. Therefore, after force-loaded gel contraction, TSP1 expression and p-ERK activation were assessed by western blotting. We found that TSP1 expression and p-ERK activation were significantly increased in force-loaded fibroblasts isolated from both normal individuals and SSc patients (Figure 4b). TSP1 expression is therefore regulated by contractile activity of fibroblasts within the three-dimensional FPCL model.

### TSP1 is induced by PDGF and TGFβ during fibroblast-mediated matrix contraction

**Figure 5:**
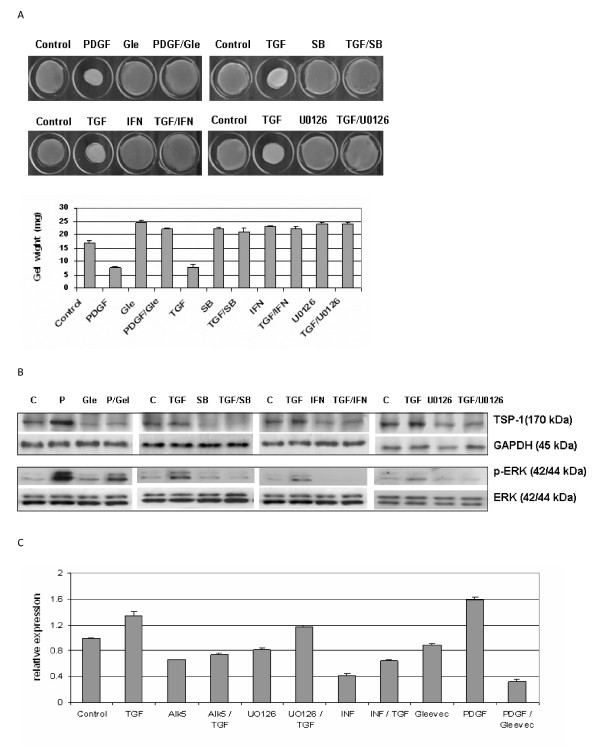
**Thrombospondin 1 (TSP1) contributed to platelet-derived growth factor (PDGF) and transforming growth factor (TGF)β-induced contractile activation in normal fibroblasts via mitogen-activated protein kinase kinase (MEK)/extracellular signal-regulated kinase (ERK) signalling pathways**. **(a) **Normal fibroblasts were treated overnight with or without TGFβ or PDGF, plus the antagonists of activin-linked kinase 5 (ALK5), ERK and PDGF, and interferon (IFN)β prior to performing a floating gel contraction assay. TGFβ-induced contractile ability was significantly reduced by IFNβ as that by SB431542 and U0126 in normal fibroblasts. PDGF-induced cell contractility was impaired by PDGF receptor inhibitor (Gleevec). **(b) **Following floating gel contraction, the fibroblasts in floating gel samples were analysed by western blotting. TGFβ-induced TSP1 expression and p-ERK activations were inhibited by SB431542, U0126 or IFNβ. PDGF induced the overexpression of TSP1 inhibited by Gleevec, which also accompanied a retrained reaction for PDGF-induced p-ERK activation in normal fibroblasts. (c) The mRNAs from the fibroblasts in these floating gel samples were also assayed by reverse transcription (RT)-PCR. The TSP1 gene expression levels altered in a similar manner to the TSP1 protein expression level within the corresponding groups.

Our previous research had demonstrated that TGFβ enhanced contractile ability of fibroblasts partly depends on ERK activation [11]. Inhibiting the TGFβ type I (ALK5) receptor reduced the contractility of fibroblasts, but not α-SMA expression and stress fibre formation [7,10]. Conversely, the PDGF/c-abl inhibitor Gleevec reduced ECM contraction and α-SMA expression [11]. Previously, it was shown that the antifibrotic effect of interferon β in lung fibrosis occurred via inhibiting TGFβ activation and decreasing TSP1/2 expression [36]. Our current data showed that TSP1 contributed to the ability of fibroblasts to contract matrix and phosphorylate ERK. To further confirm the relationship between MEK/ERK signalling pathway and TSP1 function on the contractile ability of fibroblasts, normal fibroblasts were treated for 24 h with or without TGFβ in the presence or absence of SB431542 (ALK5 inhibitor), U0126 (MEK inhibitor) or IFNβ prior to performing a floating gel contraction assay. A floating collagen gel contraction assay was used to show that TGFβ-induced contractile ability was significantly reduced by IFNβ as well as SB431542 and U0126 (Figure 5a). Following floating gel contraction, the fibroblasts in floating gel samples were analysed by western blot. Our results showed that TGFβ-induced TSP1 expression was inhibited by SB431542 (ALK5 inhibitor), U0126 (ERK inhibitor) or IFNβ. It is interesting to note that TGFβ-induced p-ERK activation also was inhibited by SB431542 and IFNβ (Figure 5b). PDGF can markedly potentiate tissue repair *in vivo *and also may stimulate cells to express growth factors such as TGFβ [36,37]. The expression of TSP1 *in vitro *can be induced by platelet-derived growth factor (PDGF) [38]. Therefore, normal fibroblasts were also treated with PDGF or the PDGF receptor inhibitor Gleevec prior to conducting collagen gel contraction assays. We found that PDGF-induced contractile ability, ERK phosphorylation and TSP1 expression in a Gleevec-sensitive fashion (Figure 5a,b). Moreover, reverse transcription (RT)-PCR analysis of mRNAs extracted from fibroblasts subjected to ECM contraction revealed that the TSP1 mRNA levels were altered in a manner paralleling our TSP1 protein analyses (Figure 5c). All these results indicated that TSP1 is induced during PDGF-mediated and TGFβ-mediated matrix contraction by normal fibroblasts.

### Overexpression of TSP1 in SSc fibroblasts is due to endogenous TGFβ and PDGF via a MEK/ERK-dependent mechanism

**Figure 6:**
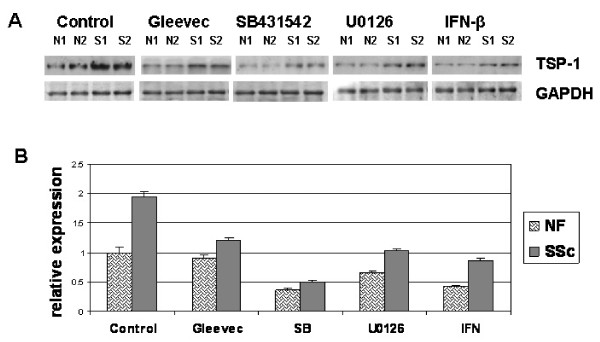
**The overexpression of thrombospondin 1 (TSP1) in systemic sclerosis (SSc) fibroblasts is dependent on endogenous transforming growth factor (TGF)β and platelet-derived growth factor (PDGF) via mitogen-activated protein kinase kinase (MEK)/extracellular signal-regulated kinase (ERK) activation**. SSc fibroblasts were pretreated with SB431542 (activin-linked kinase 5 (ALK5) inhibitor), U0126 (ERK inhibitor), interferon (IFN)β or Gleevec (PDGF receptor inhibitor) overnight, prior to determining TSP1 gene and protein expression. **(a) **Western blotting showing that SB431542, U0126, Gleevec and IFNβ inhibited the overexpression of TSP1 protein in SSc fibroblasts. **(b) **The mRNAs of fibroblasts in these treated samples were also assayed by reverse transcription (RT)-PCR.

Our previous work showed that TGFβ receptor type I (ALK5) and MEK/ERK contribute to the elevated contractile abilities of SSc fibroblasts [7,10]. Therefore, we wanted to further clarify whether the overexpression of TSP1 in SSc fibroblasts is impacted by blocking endogenous TGFβ and PDGF signalling, SSc lesional fibroblasts were treated overnight with SB431542 (ALK5 inhibitor), U0126 (MEK inhibitor) or IFNβ. TSP1 protein and mRNA expression were assayed with western blotting and RT-PCR. Our results showed that mRNA and protein expression of TSP1 in SSc fibroblasts were inhibited by antagonists of ALK5 and MEK, as well as IFNβ. SSc fibroblasts treated with Gleevec also showed reduced TSP1 mRNA and protein (Figure 6). Collectively, these results indicated that the enhanced contractile ability of SSc dermal fibroblasts depends on TSP1 induction downstream of endogenous TGFβ and PDGF through MEK/ERK. Moreover, our data provide clear evidence that TSP1 plays a key role in mediating the fibrotic phenotype observed in SSc.

## Discussion

The contraction processes in wound and fibrotic tissue mainly depend on a specialised form of fibroblasts known as myofibroblasts, which express the procontractile protein α-SMA. It is also well know that a number of integrins are responsible for cell contraction within different types of cells [39,40]. Our previous data had found that dermal fibroblasts from SSc lesions are characterised by enhanced contractile ability of SSc fibroblasts and expression of a cohort of overexpress profibrotic genes, including α-SMA and integrins [7,10]. TGFβ1 is a key factor in mediating both in fibroblasts' participation in wound repair and in a promoting pathological fibrosis, including SSc. Treatment of fibroblasts with TGFβ results in their differentiation into myofibroblasts and also stimulates their production of extracellular matrix, and adhesive proteins such as integrins [7,41]. In monolayer culture, TGFβ is partially responsible for the phenotype of lesional SSc fibroblasts [7,10]. However, it remains unclear whether activation of TGFβ signalling plays a role in ECM contraction in three-dimensional models of contraction. The data presented in this investigation shown that TSP1 is tightly linked with the enhanced contractility of SSc fibroblasts in the context of a three-dimensional culture system, as knockdown of the TSP1 gene or a blocking anti-TSP1 peptide, which prevents activation of latent TGFβ, reduced the cell contractility of fibrotic SSc fibroblasts. In parallel, antagonising TSP1 impaired expression of α-SMA, integrin α3, and integrin β5. Blocking TSP1 expression and activity also reduced the basal contractility of normal fibroblasts. We have found that endogenous TGFβ signalling contributes to the basal contractility of normal and SSc fibroblasts in three-dimensional FPCL. The results from our current report indicate that increased activation of latent TGFβ by TSP1 contributes to the overall activity of exogenous TGFβ during the process of ECM contraction in a three-dimensional culture. After mechanical loading of fibroblasts within the FPCL system, TGFβ activity and TSP1 expression were increased. All these results indicate that TSP1 contributes to the contractile ability of fibroblasts by promoting myofibroblast differentiation by TGFβ. Our data are also consistent with the notion that TSP1 is a key mediator contributing to the enhanced contractile ability displayed by lesional SSc dermal fibroblasts. In summary, blocking TSP1 may be a viable antifibrotic strategy.

The ability of TGFβ1 to induce TSP1 in fibroblasts is ERK dependent [42]. TSP1 can also induce ERK phosphorylation via β1 integrin [43]. Prior data from our laboratory have shown heparan sulfate-dependent ERK activation contributes to the enhanced contractile ability demonstrated by lesional dermal scleroderma fibroblasts [7]. Consistent with these results, in the current study we have shown that anti-TSP1 strategies not only reduced fibroblast contractility but also decreased ERK activation in fibroblasts subjected to ECM contraction and mechanical loading. We have also shown that TGFβ and PDGF-induced contractility in normal and SSc fibroblasts corresponded with elevated expression of TSP1 and ERK activation. It has been shown that TSP1 can bind and stabilise PDGF, enhancing the biological effect of PDGF in proliferative tissue repair [39]. It is interesting to note that the overexpression of TSP1, whether induced by TGFβ and PDGF in normal fibroblasts or basally in SSc lesional dermal fibroblasts, was inhibited by the MEK/ERK inhibitor (U0126). All these results indicate that, as an endogenous activator of TGFβ, TSP1 contributes to the pathological contractile activity of SSc fibroblasts. Moreover, TSP1 may also potentially mediate responses to PDGF in the pathogenesis of SSc. Our results are consistent with a previous suggestion that constitutive overexpression of TSP1 in SSc fibroblasts depends on autocrine TGFβ signalling [9].

Lesional SSc dermal fibroblasts overexpress syndecan 4, CCN2 and TSP1 [7,9,10]. CCN2 is expressed by mesenchymal cells undergoing active tissue remodelling, and is characteristically overexpressed in connective tissue pathologies such as fibrosis and cancer [6,44]. Heparan sulfate chains of syndecan 4 mediate response to growth and differentiation factors such as TGFβ [45]. Syndecan 4 also binds CCN and acts as a coreceptor for CCN2 [46]. Although the precise nature of the interactions among syndecan 4, CCN2 and TSP1 is still unclear, our previous investigations found low expression of TSP1 in fibroblasts isolated from syndecan 4 -/- or CCN2 -/- mice [11,47]. In our current study, TSP1 knockdown with siRNA did not alter expression of syndecan 4 and CCN2. Collectively, these results suggest expression of TSP1 in fibroblast culture is downstream of both syndecan 4 and CCN2. It has been reported that, in a mouse model of arthritis, injection of TSP1 blocking peptides for 16 days reduced joint infiltration and inflammation and CCN2 message and protein levels [9]. However, this reduced CCN2 could result indirectly due to the ability of TSP1 to activate latent TGFβ. Alternatively, a mechanism involving activation of cell types other than fibroblasts might be involved. Therefore, whether TSP1 directly affects CCN2 expression *in vivo *in SSc still needs to be investigated.

We have previously shown that the ras/MEK/ERK 'classical' MAP kinase cascade is important for several features of fibrogenesis. For example, MEK/ERK mediates the induction of CCN2 expression in normal mesenchymal cells [48,49]. In addition, MEK/ERK is required for α-SMA stress fibre assembly, via a syndecan 4-dependent mechanism [7,10]. Moreover, the enhanced constitutive ERK activation in lesional SSc fibroblasts is due to an increase in syndecan 4 expression [7,10]. The MEK-ERK pathway and HSPGs contribute to the overexpression of profibrotic proteins and enhanced contractile forces in SSc dermal fibroblasts, and the procontractile signals from TGFβ are integrated through syndecan 4 and MEK/ERK [11]. TGFβ has long been hypothesised to be a major contributor to pathological fibrotic diseases [41]. In this investigation we showed that TSP1-mediated TGFβ activation contributes to the pathological contractile activity of SSc fibroblasts via an ERK-dependent mechanism. In contrast, as a multifunctional cytokine, TGFβ is not only a key regulator of extracellular matrix assembly and remodelling but also affects a wide variety of cellular processes. Therefore, therapeutic strategies focusing on non-specific, systemic blockade of TGFβ ligand-receptor interactions may have a problematic side effect profile considering the complex function of TGFβ *in vivo *[41]. Conversely, TSP1 is a multicellular protein that modulates cell functions and cell-matrix interactions [50]. Abnormalities observed in TSP1-null animals resemble those observed in TGFβ1 deficient animals, but are much less severe [51]. Collectively, our results suggest that, as compared to broad targeting of TGFβ, TSP1 may be an ideal therapeutic target for fibrotic diseases such as SSc.

## Conclusions

In summary, in this report we provide useful information to further understand the mechanism underlying extra cellular matrix contraction by fibroblasts and exaggerated TGFβ signalling in the pathogenesis of SSc. Our results could also prove to be a great advantage as a potential therapy for disorders characterised by the enhanced activity of TGFβ in fibrotic disorders such as SSc.

## Competing interests

The authors declare that they have no competing interests.

## Authors' contributions

YC, AL, DJA and ME designed and conceived the study. YC, LK, XS, ME, DJA and AL performed and interpreted the experiments. CPD, CMB and LSV assisted with the experimental design and discussed the manuscript. YC, AL, DJA and ME wrote the manuscript. All authors read and approved the final manuscript.
