# Erratum to: Thrombospondin 1 is a key mediator of transforming growth factor b-mediated cell contractility in systemic sclerosis via a mitogen-activated protein kinase kinase (MEK)/extracellular signal-regulated kinase (ERK)-dependent mechanism

**DOI:** 10.1186/s13069-015-0021-1

**Published:** 2015-03-14

**Authors:** Yunliang Chen, Andrew Leask, David J Abraham, Laura Kennedy, Xu Shi-wen, Christopher P Denton, Carol M Black, Liaquat S Verjee, Mark Eastwood

**Affiliations:** School of Life Sciences, University of Westminster, London, UK; Department of Physiology and Pharmacology, Canadian Institute of Health Research Group in Skeletal Development and Remodelling, Division of Oral Biology and Schulich School of Dentistry, University of Western Ontario, London, ON Canada; Department of Inflammation, Centre for Rheumatology, University College London, London, UK; Kennedy Institute of Rheumatology, Imperial College London, London, UK

## Erratum

After publication of this work [1], the authors became aware of some errors in the figures with respect to the loading controls for the western blots in Figure Two panel A (Figure 1 here), Figure Five panel B (Figure 2 here) and Figure Six panel A (Figure 3 here). These errors were due to genuine mistakes in generating the figures from templates and incorrect cropping of the western blot X-ray film images. These errors had no impact on the scientific conclusions of the article. The experiments reported in these figures have been repeated and new images produced.

**Figure 1:**
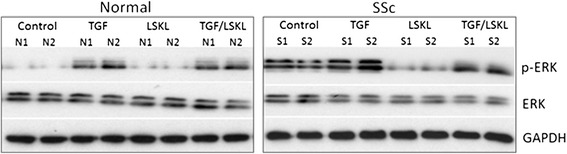
**Blocking thrombospondin-1 (TSP1) signalling with a LSKL peptide reduces ERK phosphorylation in control and systemic sclerosis (SSc) fibroblasts.** Following gel contraction in the presence of TSP1 blocking peptide LSKL (AnaSpec), fibroblast lysates were prepared for western blotting and membranes probed with antibodies against total ERK and phospho-ERK (both from Cell Signalling) and GAPDH (Abcam) as the loading control. LSKL peptide reduced the expression of phospho-ERK in SSc fibroblasts.

**Figure 2:**
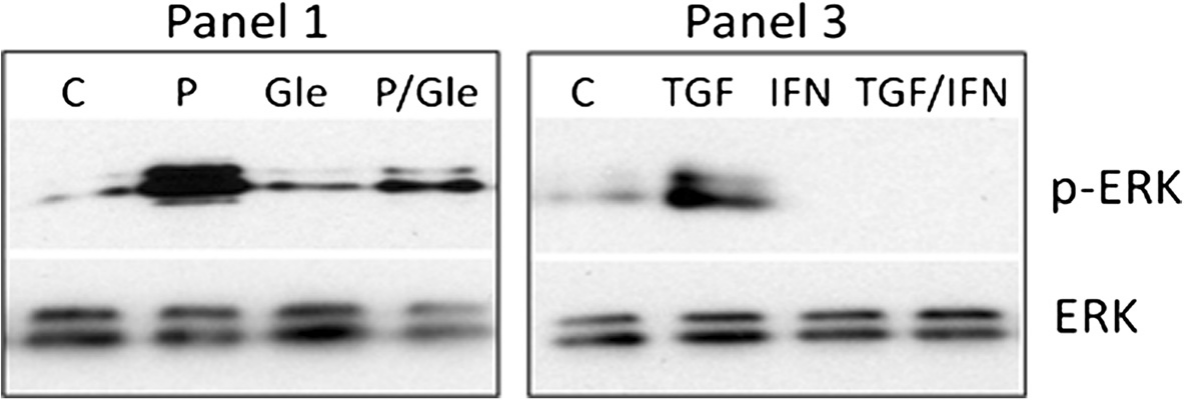
**Thrombospondin 1 (TSP1) contributes to platelet-derived growth factor (PDGF) and transforming growth factor (TGF)b induced contractile activation in normal fibroblasts via the ERK signalling pathways.** Normal fibroblasts were treated in collagen gels with or without PDGF(P), TGFb or INFb (IFN), and in the presence of PDGF and the PDGF receptor inhibitor Gleevac (Gle), or combined TGFb and INFb. Following gel contraction samples were analysed by western blotting for expression of total ERK and phospho-ERK compared to control untreated cells (C). PDGF and TGFb induced the levels of phospho-ERK, which were inhibited in the presence of Gleevac and IFNb respectively.

**Figure 3:**
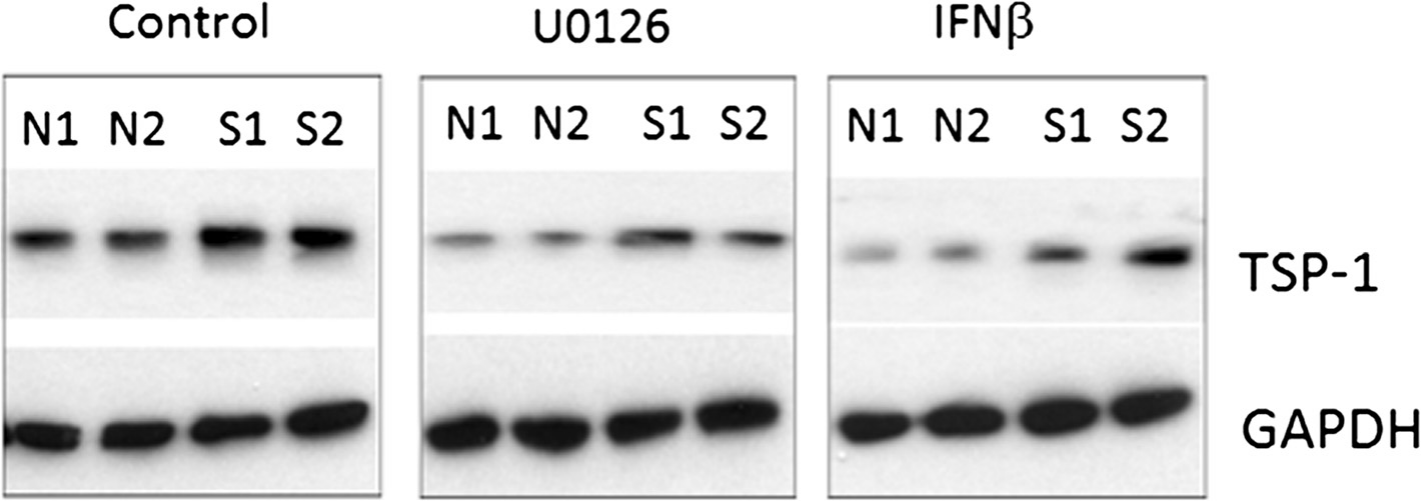
**The overexpression of thrombospondin 1 (TSP1) in systemic sclerosis (SSc) fibroblasts is dependent on endogenous activation of ERK.** Normal and SSc fibroblasts were treated with U0126 (ERK inhibitor) and interferon (IFN)b overnight, prior to determining protein expression by western blotting using an anti-TSP1 antibody (Abcam). U0126 and IFN inhibited the over expression of TSP1 protein in SSc fibroblasts.

Figure Two panel A (Figure 1 here) - Experiment assessing the influence of blocking thrombospopndin- 1 on normal and scleroderma fibroblasts in 3-dimensional collagen gels was performed and western blot for loading control GAPDH as well as total ERK and phospho-ERK were completed.

Figure Five panel B (Figure 2 here) - Role of PDGF, IFNb and TFGb and the influence of the kinase inhibitor Gleevac on MAPK activation by normal fibroblasts was carried out and the levels of total ERK and phospho-ERK assessed.

Figure Six panel B (Figure 3 here) - The effect of the ERK inhibitor (U0126) and IFNb on the expression of thrombospondin-1 compared to the loading control (GAPDH) was re-examined in normal and scleroderma fibroblasts.

## References

[1] Yunliang C, Andrew L, Abraham DJ, Laura K, Xu S-w, Denton CP (2011). Thrombospondin 1 is a key mediator of transforming growth factor b-mediated cell contractility in systemic sclerosis via a mitogen-activated protein kinase kinase (MEK)/extracellular signal-regulated kinase (ERK)-dependent mechanism. Fibrogenesis and Tissue Repair.

